# Current Research on the Use of the Omental Flap in Breast Reconstruction and Post-Mastectomy Lymphedema: A Focus on Omental-Vascularized Lymph Node Transfer

**DOI:** 10.3390/life13061380

**Published:** 2023-06-13

**Authors:** Farrah C. Liu, Kometh Thawanyarat, Yelissa Navarro, Dung H. Nguyen

**Affiliations:** 1 Division of Plastic & Reconstructive Surgery, Stanford University, Stanford, CA 94304, USA; 2 Medical College of Georgia, Augusta University, Augusta, GA 30912, USA; kthawanyarat@augusta.edu (K.T.); ynavarro@augusta.edu (Y.N.)

**Keywords:** breast, reconstruction, omentum, omental flap transfer, lymphedema, plastic surgery

## Abstract

The novel use of the omental flap in breast reconstruction has been increasing in research popularity within the last few decades. This technique has its roots in the early 20th century as surgeons explored the use of the omentum for a variety of reconstructive purposes across various surgical subspecialties. The current literature shows evidence of the benefits of using the omentum in autologous breast reconstruction compared to the more traditional abdominal, flank, thigh, and gluteal donor flap reconstruction. This method introduces a viable option for patients that do not meet the criteria for the traditional autologous reconstruction techniques allowing for the restoration of more natural appearing breasts without the added complication of donor-site mortality. Additionally, the omentum, with its rich source of vascularized lymph nodes, has been studied as a potential source for lymph node transfer in the treatment of mastectomy-associated lymphedema. In this review, we highlight the most recent research on the current practices of omental-based breast reconstruction techniques and their use in postmastectomy lymphedema. We discuss the history and natural progression of the development of omental-based reconstruction as an autologous breast reconstruction technique, highlight the latest advances and challenges for the utility of the omental flap in current surgical procedures, and present future directions for the potential role of omental-based breast reconstruction in postmastectomy breast surgery.

## 1. The History of the Omental-Based Breast Reconstruction

The use of the omental flap in reconstructive surgery has been observed in various specialties for its rich vascularity and tissue regeneration properties [1,2]. In plastic and reconstructive surgery, omental flaps have helped innovate the fields of limb salvage revascularization, osseous reconstruction, and wound defects providing extensive coverage and adequate blood flow to sustain healing, replace and reform interrupted vasculature, and supply unique physiological functions that promote improved survival and infection control [3,4,5]. The nature of the omentum makes it a versatile option for autologous reconstruction surgery [6,7]. The omentum, which arises from the most anterior portion of the abdominal cavity, takes on many physiological roles in the body [7]. The omentum is composed of fatty tissues, arteries, veins, and lymphatics systems, all contributing to its remarkable and multi-faceted role as a functioning organ of the body [5,6,7]. Omental tissue size may reach upwards of 2000 g with a surface area of up to 1500 cubic centimeters [6,8]. While its composition is largely that of adipose tissue—providing a source for fat storage readily accessible and available—its function extends to cover regeneration, revascularization, immune regulation, hemorrhage control, and scar formation to stifle possible trauma sustained by the visceral organs that lay posterior to the structure [7,8]. Blood supply to the structure relies on the gastroepiploic arteries branching from the gastroduodenal and splenic arteries along the border of the greater curvature of the stomach [6]. As an organ, the omentum has been shown to play a vital role in peritoneal protection both anatomically and physiologically [1,6,7]. However, it is its neovascularization, tissue healing, and tissue regenerative properties that have led to many innovators in various fields of surgical medicine to harvest the reconstructive support of the organ [1,2,3,6,7,9,10].

The current use of the omentum in surgery extends to the specialties of general surgery, thoracic surgery, neurosurgery, vascular surgery, orthopedic surgery, urogenital surgery, and gynecologic surgery [7]. Specific to plastic surgery, the omental flap has been a source of donor tissue that facilitates the reconstruction of various anatomical locations including the head and neck, the extremities, locally in the abdominal cavity, and in various cases of advanced breast cancer [6,7,11]. In terms of breast cancer reconstruction, omental-based breast reconstruction was first described as an alternative to autologous reconstruction from the more traditional donor sites of the abdomen and thigh by Kirikuta in 1963 [9,10]. Since then, the field of omental-based breast reconstruction has significantly broadened, emerging as an effective alternative for autologous breast reconstruction options [10].

The autologous method for breast reconstruction has been determined by researchers throughout the field as providing comparable benefits and positive long-term outcomes with more natural results compared to implant-based breast reconstruction which can often result in firmer, less natural breast mounds and increase the risks associated with foreign material in the body [10]. The traditional method of using the abdomen, thigh, or gluteus region as a donor site has been a suitable, if not preferred, option for patients undergoing unilateral or bilateral non-implant-based reconstruction. By utilizing the rectus abdominus, latissimus dorsi, transverse or diagonal upper gracilis, gluteal, or any other appropriate muscle flap with transposable vasculature, surgeons are able to recreate a warm, soft, and natural breast mound in the place of removed breast tissue and skin that may be necessary for patients with locally involved breast cancer [8,9,10,12]. However, the benefit of utilizing this approach relies on suitable patient factors prior to choosing autologous reconstruction as an option. This method requires the availability of sufficient tissue at the donor-site location which may not be possible for patients with a lower body mass index (BMI) [13]. Additionally, patients who have had prior surgeries such as abdominoplasty or thighplasty, or those patients not willing to risk donor-site complications may find that the autologous reconstruction approach is not an adequate option for their breast cancer therapy [8,13]. While implant-based reconstruction is the most commonly used technique when considering breast reconstruction, advances in autologous reconstruction have increased the choices available for patients and their surgeons to more carefully decide the course of surgery that fits their needs and safety profile—i.e., considerations in cases of radiation after mastectomy [14,15].

## 2. Autologous Breast Reconstruction versus Implant-Based Breast Reconstruction

Autologous reconstruction has proven to be a worthy alternative to implant-based reconstruction when considering patient satisfaction, long-term results, and complications, including implant failure or flap loss [12,16,17,18]. A study by Garvey et al. found that reconstruction by alloplastic methods results in higher failure rates compared to muscle or tissue flaps [16]. Additionally, the two approaches show no differences in outcome when comparing immediate reconstruction to delayed reconstruction with implant cases trending towards higher failure rates [16]. In a literature review spanning over 200 studies, Toyserkani et al. found that satisfaction rates were higher across studies for patients that received autologous-based breast reconstruction compared to implant-based reconstruction when evaluating psychosocial, sexual, and overall outcomes, further strengthening the rationale for presenting the option of autologous reconstruction to potential patients considering reconstruction post-mastectomy [17]. A study by von Glinski et al. reflected similar findings showing that autologous reconstruction not only presents with higher patient-reported satisfaction ratings but also greater aesthetic outcomes [18].

When studying complication rates among autologous breast reconstruction compared to implant-based reconstruction, many studies present varying rates of major and minor complications [18,19,20]. von Glinski et al. found that implant-based reconstruction resulted in a greater number of major complications—including implant malposition, major infection, capsular contracture, and need for revision surgery [18]. The same study found no significant difference in the incidence of minor complications—including hematoma, seroma, minor wound infections, and wound healing disorders—which have been seen in the previous literature to trend upwards in patients undergoing autologous breast reconstruction [18]. For instance, Bennet et al. with similar results did note a significant finding in minor complications among the implant-based versus autologous reconstruction showing that minor complications—including hematoma, seroma, minor wound infections, and wound healing disorders—were more common among autologous breast reconstruction [19]. Despite the increased occurrence rates, these minor complications rarely lead to flap loss in autologous reconstruction [18,19,20]. Major complications—as mentioned previously—were less often seen than minor complications in cases of autologous reconstruction and are more common among implant-based reconstruction [18,19,20]. The major complications are also more likely to lead to reconstruction failure requiring implant explantation for implant-based reconstruction [19,20]. So while autologous reconstruction carries a greater likelihood of minor complications, the complications usually require minor resolution and rarely lead to major revision surgery or flap failure. In contrast, implant-based reconstruction has fewer complications, though the complications that do arise are more likely to lead to revision surgery and explanation [18,19]. In addition, certain implant-based complications, such as rupture and capsular contractures, are not seen in autologous reconstruction [17,18]. Other major complications, such as infection, may necessitate more aggressive intervention for implant-based reconstruction—i.e., implant explantation and washout—that is not typically indicated for autologous reconstruction procedures as there is no foreign material [19]. As far as minor complications, results have shown comparable outcomes in terms of patient satisfaction after long-term studies, which revisits the position that either option is suitable for breast reconstruction [18].

Across studies, the patient-centered benefits of the use of autologous-based breast reconstruction have shown to be comparable and may at times surpass the expected overall outcomes and long-term satisfaction rates compared to implant-based reconstruction. Moreover, complications unique to implant-based use, such as Breast Implant Associated Anaplastic Large Cell Lymphoma (BIA-ALCL), has made autologous reconstruction a more palatable option with less significant and life altering complications. Moreover, whether or not complications arise in implant-based reconstruction, the increased risk of implant failure over time and the need for implant replacement may require consideration for future surgical planning that is not necessary in autologous-based breast reconstruction. Finally, for certain patients, the allure of using their own tissue to reform their breast may be an additional factor that influences the decision towards autologous reconstruction which would be able to provide them with their desired outcomes and improved quality of life.

## 3. Current Practices and Methods of Omental-Based Breast Reconstruction

### 3.1. Omental-Based Reconstruction as an Alternative Method in Autologous Reconstruction

The current techniques of autologous-based breast reconstructions, including latissimus dorsi flap, TRAM (transverse rectus abdominis muscle) flap, DIEP (deep inferior epigastric perforator) flap, SIEA (superior inferior epigastric artery) flap, superior gluteal artery perforator (SGAP) flap, inferior gluteal artery perforator (IGAP) flap, transverse or diagonal upper gracilis (TUG/DUG) flaps, and profunda artery perforator (PAP) flap have an established and robust history for effective breast reconstruction, showing greater long-term patient satisfaction when compared to implant-based reconstruction [12,17]. The various benefits for the use of autologous reconstruction include long-lasting results, the ability to age naturally with the patient, and responsiveness to fluctuations in patient weight or other body habitus [21]. The autologous reconstruction approach is particularly beneficial for overweight or obese patients with improved outcomes compared to implant-based reconstruction [16]. However, autologous breast reconstructions from abdominal, thigh, gluteal, or dorsal flaps may not be an option for all patients. Patients lacking sufficient skin or myocutaneous tissue at the donor site, those unwilling to risk donor-site injury, or those with a previous injury or surgery at the donor site may not have the option to utilize traditional tissue flaps in their reconstruction. These patient considerations highlighted the need to find alternative sources of abundant tissue that could be utilized to reform a patient’s breast. With its numerous physiological properties, many researchers in the field of plastic surgery considered the use of the omental flap as a source of tissue able to provide an alternative technique for autologous reconstruction [6]. Initially used to treat congenital breast deformities, the use of the omentum has expanded to include reconstruction after lumpectomy and radiation, with the progression of omental-based autologous reconstruction shifting to its utilization in partial and total breast reconstructions [21,22,23]. For breast tissue damage secondary to radiation, omental-based breast reconstruction has resulted in exceptional outcomes that take advantage of the nature of the omentum to promote the regeneration and healing of damaged tissues [21]. This approach has ultimately resulted in promising outcomes such as natural-looking breasts, increased patient satisfaction, minimal scarring, and low risk of deformity at the donor site [24,25,26]. Over time, the switch of procurement methods from an open to laparoscopic technique has made omental-based breast reconstruction a minimally invasive and safe procedure rarely resulting in laparoscopic complications—such as GI perforations, incisional hernia, or abdominal wall hemorrhage [27,28,29,30]. Omental-based breast reconstruction has been shown to be a suitable alternative option in autologous reconstruction approaches and may be the only option for a number of select patients hoping to benefit from the numerous advantages of the omentum.

### 3.2. Omental Fat-Augmented Free Flap Reconstruction

Current methods for autologous reconstruction involving free or pedicled myocutaneous tissue flaps have provided favorable outcomes for patients undergoing non-implant-based breast reconstruction. However, the requirements for free or pedicled flaps such as the latissimus dorsi, TRAM, DIEP, or SIEA flaps may make the option not appropriate or attainable for patients with a lower BMI or inadequate adipose tissue volume [13,31]. Additionally, patients opting for bilateral breast reconstruction that requires more tissue volume to appropriately reconstruct a breast mound ideal for their body frame may have to forgo the benefits of autologous reconstruction and may only have the option for implant-based reconstruction. While implant-based reconstruction is a comparable choice when considering long-term patient satisfaction, this option limits patients that may desire a non-implant-based reconstruction if they do not meet the appropriate criteria [8,31]. Omental fat-augmentation free flap (O-FAFF) reconstruction has been tested as an innovative technique to solve this problem, increasing the population of patients that can be considered candidates for autologous reconstruction by using a combination technique of omental flap harvesting in addition to fat harvesting to increase omental flap volume [8,23,31].

**Figure 1:**
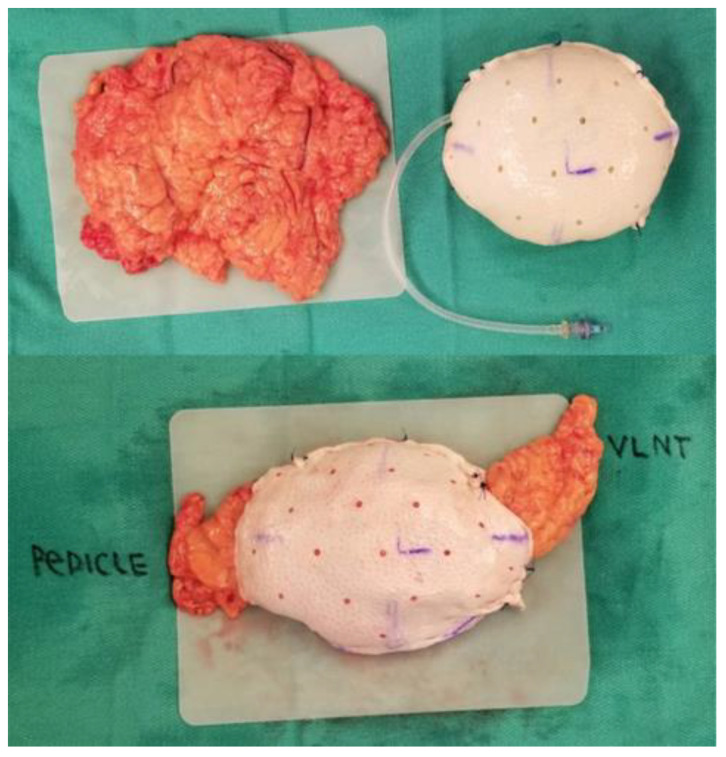
A breast sizer is used to tailor the acellular dermal matrix pocket. The gastroepiploic pedicle is delivered out through a lateral opening. In this breast construct, an additional slip of omentum was brought out on the opposite site to be used as a VLNT to the axilla at the time of complete axillary lymph node dissection [32].

The technique as described by Nguyen et al. begins after mastectomy, either nipple sparing or skin sparing, and it involves laparoscopic omentectomy that can be performed simultaneously with the mastectomy and ex vivo suturing of an acellular dermal matrix (ADM) mold to hold the harvested omental adipose tissue [31]. While previous abdominal surgery can present with certain risks for omental procurement, history of surgery is not an absolute contraindication. The greatest risk in these reconstruction cases involves maintaining adequate blood flow to the omentum which can be confirmed by computed tomography (CT) angiography [31]. After clipping the vasculature and dissecting the borders of the omentum along its connection, the organ is removed along with its vasculature with the option to divide the tissue ex vivo if necessary for bilateral reconstruction. Fat is then harvested for omental augmentation by liposuction of the bilateral thighs and abdomen until reaching the desired size of the omental flap. The harvested fat is grafted directly into the omental adipose tissue under direct visualization to avoid injury to the large omental vessels using the Coleman lipo-injection technique. The ratio of fat to omentum weight used ranges from 0.22 to 1.38 for unilateral cases and 0.24 to 3.8 for bilateral cases. The harvested omentum is then fashioned into an organic implant covered in an acellular dermal matrix (ADM) mold that is trimmed and tailored around a breast sizer that is selected based on the patient’s breast base width with an opening in the medial aspect of the mold for anastomosis of the omental vessels (Figure 1). Microsurgical anastomosis of the omentum via the gastroepiploic vessels to the internal mammary vessels in a post-mastectomy breast pocket provides adequate blood flow for the flap. The flap is then monitored postoperatively with transcutaneous dopplers [31]. Recovery for omental-based breast reconstruction is largely unchanged from the more traditional autologous reconstruction methods with improvement in pain management and less use of narcotics compared to the control group [31].

The novel technique of O-FAFF presents many advantages in the use of omental-based breast reconstructions. These advantages include less donor-site morbidity and fewer complications—including abdominal wall weakness, postoperative hernias, and scarring—which are among the greatest concerns in the use of traditional autologous breast reconstruction. In addition, the less invasive flap harvest via laparoscopic approach presents with lower scoring of pain, shorter hospital stays, and faster recovery time after the procedure [31,33]. These complications are further augmented in cases requiring bilateral reconstruction. This technique overcomes the main limitations of the use of omental flap in the volume acquired for reconstruction. Augmentation via fat transfer to the omentum demonstrates the potential resolution for low-volume harvesting and helps restore a patient’s natural breast volume [31].

### 3.3. Concurrent Treatment for Lymphedema: Omental Vascularized Lymph Node Transfer

The emergence of omental-based reconstruction has subsequently brought attention to more uses for the omentum in post-mastectomy complications. The use of omentum in the treatment of lymphedema has also been highlighted with the rise of omental-based harvesting techniques. One of the possible complications for breast cancer patients is the development of post-mastectomy lymphedema following treatment secondary to axillary dissection, sentinel lymph node biopsy, and radiation therapy. Lymphedema is a debilitating disease that requires constant maintenance therapy and may be amenable to surgical intervention including lymphovenous bypass (LVB), lymphaticovenous anastomosis (LVA), or a vascularized lymph node transfer (VLNT) [32]. Lymphedema can then continue, causing progressive injury to the affected tissue that may eventually result in permanent fibrosis. One of the more suitable surgical interventions introduced to attempt to halt the progression of the disease is VLNT. Different VLNTs have been described in the literature with lymph node sources including inguinal, gastroepiploic, thoracic, supraclavicular, submental, and omental [32]. Due to its success in the treatment of ipsilateral upper extremity lymphedema, omental vascularized lymph node transfer has increased in popularity to reduce the risk of donor-site lymphedema when compared to other similar recipient sites.

Goldsmith et al. first described utilizing the omentum for the treatment of lymphedema in the 1960’s [34,35]. The researchers detailed the transposition of the omentum for extremity lymphedema with clinical success. However, the obvious disadvantages in procurement involved the required laparotomy for harvest that can increase complications at the donor site, as well as the potential complications with the pedicle lymph node. In 1979, Shesol et al. reported the first VLNT after it was successfully transferred in an animal model [36]. The authors described the transfer of autologous vascularized lymph nodes into the site of lymphedema as perhaps working through lymphangiogenesis or allowing the transferred nodes to act as a lymphatic pump [36]. In 1994, Ergorov et al. described the omentum as a free revascularized omental graft for lymphedema treatment, decreasing the risk of compromising the blood supply and lymphatic channels that are associated with the pedicled omentum with results showing improvement of swelling in 19 out of 21 patients [37].

Since its initial discovery, several studies have shown the success of laparoscopically harvesting the omentum which aims at reducing the complications associated with open harvesting including infection, visceral injury, and GI involvement [32]. In 2015, Nguyen et al. described a novel approach for the laparoscopic harvest of the omentum with microvascular free tissue transfer for the treatment of post-mastectomy-associated lymphedema [38]. Since then, there has been an increasing number of studies emerging showing that vascularized omental lymphatic transfer is safe and efficacious. In addition to minimizing the morbidity of the laparotomy through laparoscopic harvesting techniques, there is no risk of donor-site lymphedema with increased availability of lymph node tissue available for transplantation [32]. The unique immunogenic properties of the omentum also add to the benefits of its utility in VLNT.

**Figure 2:**
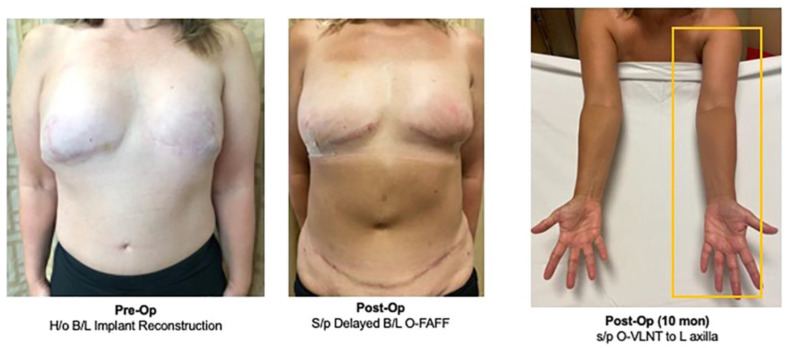
Pre- and postoperative results after delayed bilateral breast reconstruction with bilateral O-FAFF and simultaneous O-VLNT to left axilla. She maintained normal volume in the left arm at 10 months follow-up [32].

The advantages of the use of the omental flap for both autologous breast reconstruction and the treatment of lymphedema have clearly been demonstrated as a beneficial option for the treatment of breast cancer patients (Figure 2). Different techniques using latissimus dorsi flaps, inguinal flaps, gastroepiploic flaps, muscle-sparing transverse rectus abdominis muscle (MS-TRAM) flaps, and lateral thoracic VLNT with simultaneous deep inferior epigastric artery perforator (DIEP) flaps for autologous breast reconstruction have been described. These studies have reported various overall reductions in complications including a reduction in limb circumference, decreased incidence of cellulitis, and improved patient satisfaction. However, there are increased rates of complications with these methods leading to donor-site lymphedema, particularly with the harvesting of the inguinal nodes with the DIEP flap. Additionally, it can be split into multiple flaps for simultaneous autologous breast reconstruction with VLNT or for multiple sites of VLNT.

## 4. Current Challenges in Omental-Based Breast Reconstruction

Omental-based breast reconstruction can present its own set of challenges, limiting its use for certain patient cases. The nature of the omentum can make it particularly difficult to estimate tissue volume and evaluate the amount available through preoperative imaging [23]. As such, bilateral reconstruction may be limited by the amount of tissue obtained, though the novel O-FAFF technique attempts to mitigate these limits. Complications seen during omental-based autologous reconstruction are not unlike other autologous reconstruction procedures which include flap ischemia, infection, and fat necrosis [22,29]. Furthermore, the unique complications involving and affecting abdominal organs—such as increased incidence of hernia or damage to abdominal viscera—though rare, may present with their own set of limitations for certain patient populations [21]. Without a skin paddle, there are also fewer data points when clinically monitoring the omental flap during the postoperative course. The need for skin coverage also requires that patients opting for omental-based breast reconstruction can do so only if they have adequate skin coverage with enough skin over the anterior chest to cover the bulk of the tissue. Patients with involvement of the skin overlying the breast may not be a good candidates for this breast reconstruction option. Additionally, certain patient factors may result in omental-based reconstructions being contraindicated—including a history of abdominal disease, previous abdominal surgery, or immunosuppression [29]. One of the greatest challenges in the procedure of omental-based breast reconstruction stems from the anatomical structure of the omentum. The flat and malleable nature of the omentum introduces limitations in aesthetic contouring during reconstruction if not performed by an experienced reconstructive surgeon. This robust experience in omental-based reconstruction is necessary to protect the integrity of the breast structure by preventing rippling, a common consequence of omental-based breast reconstruction [29]. Experience in omental-based breast reconstruction is also paramount to decrease the risk of damage to the flap during excision with further difficulty in shaping the additional tissue to form a natural-looking contour for the breast reconstruction [23,31]. Despite these challenges, the history and progression of omental-based breast reconstruction have shown that the approach is proven to be an effective, safe, and ideal technique for autologous breast reconstruction [8,22,23,30].

## 5. Conclusions

The use of an omental flap for reconstruction has increased significantly over time with a large volume of reported cases in the last two decades [39]. The choice for an omental flap over the more traditional donor flap locations has been established, not only on the basis of patient tissue availability, but also the consideration for the risks seen in certain autologous flaps including aesthetic defects of the donor and recipient sites, muscle atrophy, and functional impairment leading to a decrease in quality of life [40]. Initial methods of omental flap harvesting involving open laparotomy present their own set of risks and complications including donor-site scarring, scarring, and donor-site morbidity [26,31,39,40]. As the initial methods for omental flap harvest evolved from open laparotomy to laparoscopic techniques in recent years, studies have seen a decrease in the risks of open procedures including damage to abdominal organs, herniations, and surgery-associated infections commonly seen in open procedures [39,40]. Currently, the laparoscopic approach has been the most widely used method for omental harvest with upwards of 80% of cases, for both free and pedicled flaps, with a decrease in complication rates overall [39]. Certain limitations of the omental flap, such as its flat structure, have led to the development and introduction of new innovative methods to provide structure and stability to the breast for a more natural breast contour, such as the O-FAFF technique [31]. With the formation of an acellular dermal matrix shell, the grafted fat from the omentum can be formed into the necessary dimensions needed to produce the most aesthetic outcomes for the patient [31].

The omental-based breast reconstruction is proven to be an effective, safe, and ideal choice for patients who desire autologous breast reconstruction post-mastectomy. The use of the omentum increases the potential patient pool that may otherwise not be candidates for autologous reconstruction and provides these patients with natural-looking, long-lasting results and successful outcomes that may present as an advantage over implant-based and other autologous-based reconstruction techniques. Furthermore, the factors that enable the omentum to be an exceptional option for tissue reconstruction also highlight its utility in the treatment of other breast-associated medical conditions. In mastectomy-associated lymphedema, the use of the omentum as a VLNT during autologous breast reconstruction allows for simultaneous treatment of lymphedema while utilizing a single donor site and minimizing donor-site lymphedema development. The roles of the omentum in various therapies are not an exhaustive list and continue to grow as more innovative uses emerge throughout reconstructive surgery. The novel role of omental-based breast reconstruction provides several approaches for therapy of conditions that commonly affect post-mastectomy patients that can potentially lead to increasing the number of patients able to undergo autologous reconstruction.
